# Dual-Mode Native Mass Spectrometry Screening Identifies Ginsenoside Ligands of 6-Hydroxymethyl-7,8-Dihydropterin Pyrophosphokinase (HPPK)

**DOI:** 10.3390/molecules31122065

**Published:** 2026-06-12

**Authors:** Xinru Xue, Ronald J. Quinn, Bernd H. A. Rehm, Peter J. Myler, Miaomiao Liu

**Affiliations:** 1 Institute for Biomedicine and Glycomics, Griffith University, Southport, Gold Coast, QLD 4215, Australia; xinru.xue@griffithuni.edu.au (X.X.);; 2 Centre for Cell Factories and Biopolymers, Institute for Biomedicine and Glycomics, Griffith University, Southport, Gold Coast, QLD 4215, Australia; 3 Center for Global Infectious Disease Research, Seattle Children’s Research Institute, Seattle, WA 98109, USA; 4 School of Environment and Science, Griffith University, Nathan, Brisbane, QLD 4111, Australia

**Keywords:** ginsenosides, 6-hydroxymethyl-7,8-dihydropterin pyrophosphokinase, tuberculosis, protein–ligand interactions, native mass spectrometry

## Abstract

Identification of ligands targeting essential enzymes in *Mycobacterium* species remains an important strategy for anti-tuberculosis drug discovery. Here, a native mass spectrometry approach was employed using pooled 100-compound mixtures, enabling the direct detection of intact HPPK–ligand complexes in solution. Dual-mode MS acquisitions (low collision energy for complex detection and high collision energy for ligand confirmation), combined with an automated data analysis workflow, ensured robust identification of binding events from these complex samples. This strategy led to the identification of several HPPK-binding small molecules, all belonging to the dammarane triterpene glycoside (ginsenoside) class. Subsequent analysis of the hits revealed clear structure–affinity relationships, highlighting how specific aglycone modifications and glycosylation patterns influence binding to HPPK. Our findings expand the known chemical space of HPPK ligands and demonstrate the utility of native MS-based screening coupled with automated data analysis to uncover new ligand scaffolds for challenging enzyme targets.

## 1. Introduction

Tuberculosis (TB) remains a global health emergency [1]. In 2024 alone, an estimated 10.7 million new cases of TB were recorded worldwide, with approximately 1.23 million deaths, making *Mycobacterium tuberculosis* (*Mtb*) the leading cause of mortality from a single infectious agent [2]. The rise of multidrug-resistant TB (MDR-TB) has further underscored the urgent need for novel therapeutic strategies beyond the current first-line drug regimen [3,4]. A key component of these efforts is the identification of new drug targets in *Mycobecterium* species and the discovery of new chemical scaffolds capable of modulating those targets [5,6].

One promising area of focus is the folate biosynthesis pathway in *Mycobacterium*, which is essential for bacterial growth and absent in humans [7,8]. Traditional antifolate drugs (e.g., sulfonamides targeting dihydropteroate synthase and trimethoprim targeting dihydrofolate reductase) have been effective antibiotics, but emerging resistance has limited their use in modern TB therapy [9,10]. 6-Hydroxymethyl-7,8-dihydropterin pyrophosphokinase (HPPK), encoded by the *folK* gene (Rv3606c), catalyzes the ATP-dependent transfer of a pyrophosphate group to 6-hydroxymethyl-7,8-dihydropterin [11,12,13]. Like other upstream folate enzymes, HPPK is essential to *Mtb*’s viability yet has no human counterpart, making it an appealing selective drug target [14,15]. Despite this, *Mtb* HPPK and related upstream enzymes have so far remained underexplored relative to the well-studied downstream targets DHPS and DHFR [16,17]. Targeting such a unique enzyme could circumvent existing resistance mechanisms and expand the arsenal against TB [18].

In the search for new inhibitors or ligands of HPPK, natural products present a rich source of chemical diversity [19]. Natural product libraries offer a wide variety of complex scaffolds that differ markedly from synthetic libraries and may reveal novel binding chemotypes [20,21]. However, conventional HTS of pure compound libraries typically tests one compound per well using an indirect functional readout, a process that is relatively slow, resource-intensive, and prone to ambiguous outcomes or false signals due to assay interference or nonspecific compound effects [22,23].

Affinity selection mass spectrometry (ASMS) has addressed some of these limitations by enabling pooled-library screening and MS-based ligand identification, and it is now a well-established strategy for ligand discovery [24]. In typical ASMS workflows, protein–ligand mixtures are subjected to separation steps to isolate bound species, followed by identification of the associated ligands. While highly effective for identifying binders from complex mixtures, this workflow involves multiple processing steps, and binding is indirectly inferred from the presence of retained ligands after separation.

Native mass spectrometry (native MS) is a label-free, fast, and accurate method that permits the direct observation of non-covalent and covalent protein–ligand complexes [25]. The technique relies on non-denaturing electrospray-ionization (ESI) to recognize multi-charged proteins in their near-native states and is fast emerging as an advanced structural biology tool in drug discovery [26]. In the resulting mass spectrum, the difference between the mass-to-charge ratio (*Δ m*/*z*) for the protein–ligand complex and the unbound protein ions multiplied by the charge state (*z*) directly affords the molecular weight of the bound ligand (MW_ligand_ = *Δ m*/*z* × *z*). The specificity of the interaction allows the molecular weight (MW) determination of ligands in complex mixtures, such as natural product extracts/fractions [27,28], pooled compound libraries [29] and pooled fragment libraries [30].

Beyond hit identification, native MS provides direct information on binding stoichiometry, complex heterogeneity, oligomeric states, and relative binding affinities [31]. Native MS can also provide estimates of apparent binding affinities by quantifying the relative abundances of apo and ligand-bound protein species observed in the mass spectrum. For protein–small molecule interactions, it is generally assumed that ligand binding does not substantially alter the ionization behavior of the protein, allowing relative signal intensities to serve as an approximation of binding populations in solution [32]. Under these assumptions, native MS has been widely applied for affinity measurements and ranking of ligand-binding strengths [33]. Recent developments in instrumentation, such as ultra-high mass range Orbitrap platforms [34], ion mobility spectrometry [35], and surface-induced dissociation [36], have further expanded the analytical capability of native MS for the characterization of increasingly large and heterogeneous biomolecular assemblies.

To date, the majority of native MS ligand discovery studies have focused on soluble proteins, including enzymes, transcription factors, viral proteins, and protein–protein interaction targets, owing to their relatively straightforward sample preparation and gas-phase stability. However, significant progress has recently been made in extending native MS to membrane proteins, which remain highly challenging targets in drug discovery [37,38]. Advances in detergent selection [39], amphipols [40], nanodiscs [41], and other membrane mimetic systems have enabled the preservation and transfer of intact membrane protein complexes into the gas phase while retaining bound lipids and ligands. These developments have facilitated native MS studies of G protein-coupled receptors (GPCRs) [42,43], ion channels [44,45], and other pharmaceutically important membrane proteins, allowing direct interrogation of ligand binding, lipid interactions, allosteric modulation, and conformational stability.

Native MS has also emerged as a valuable platform for studying higher-order protein assemblies and dynamic interactions in increasingly complex biological systems [46]. Applications now include characterization of PROTAC-mediated ternary complexes [47,48], protein–nucleic acid assemblies [49], intact viral particles [50], and endogenous ligand binding directly from partially purified biological samples [51,52]. Together, these advances position native MS as a versatile and rapidly evolving technology for ligand discovery and mechanistic investigation across a broad range of therapeutic targets.

In this study, we applied a high-throughput native MS screening approach to identify potential ligands of *Mycobacterium smegmatis* HPPK from a large, structurally diverse natural product collection. *M. smegmatis* HPPK was used as a non-pathogenic surrogate enzyme for initial hit finding because it is an Rv3606c ortholog with substantial sequence conservation to *M. tuberculosis* HPPK, with approximately 62% amino-acid identity and 73% positive similarity over aligned residues (Figure S1) [53]. Importantly, key catalytic residues and active-site features of HPPK are conserved between the two enzymes, supporting preservation of the catalytic mechanism and ligand-binding environment.

By directly observing HPPK–small molecule complexes under physiologically relevant conditions, we aimed to uncover previously unrecognized ligand scaffolds for HPPK and gain insight into the structural features that govern these binding interactions. This target-based, label-free screening strategy allowed us to rapidly highlight promising HPPK binders for further characterization, demonstrating an effective route to probe the ligandability of a challenging anti-TB enzyme target.

## 2. Results

### 2.1. A Dual-Mode Native Mass Spectrometry (MS) Workflow

**Figure 1:**
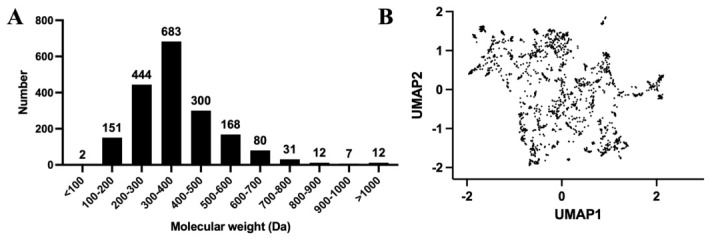
(**A**) Molecular weight distribution of the natural product library; (**B**) Structural similarity map of the screened natural product library generated from RDKit Morgan fingerprints (ECFP4, radius = 2, 2048 bits) and visualized by UMAP using Tanimoto/Jaccard distance. Each black dot represents one compound.

To identify ligands targeting HPPK, a native MS-based screening approach was applied to an in-house natural product library comprising 1890 compounds from extensive isolation efforts [54,55,56]. The library consists of structurally diverse natural products, predominantly within a molecular weight range of 200–800 Da (Figure 1A). The chemical diversity of the library was further evaluated using a Morgan fingerprint-based UMAP projection (Figure 1B) [57,58]. Morgan fingerprints encode molecular substructures and connectivity patterns into numerical representations, enabling structural similarity analysis across large compound datasets [59]. Dimensionality reduction by UMAP revealed a broad distribution of compounds throughout the chemical space, indicating substantial structural diversity within the library and supporting its suitability for ligand discovery screening. Compounds were randomly grouped into 19 pools (up to 100 compounds per pool). Pools containing fewer compounds were supplemented with DMSO to maintain constant volume and uniform ligand concentrations across all pools.

**Figure 2:**
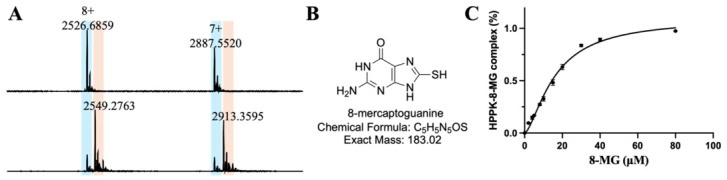
(**A**) Native MS spectra of *M. smegmatis* HPPK (1 μM) in the absence (top) and presence of 8-mercaptoguanine (8-MG, 10 μM). Apo HPPK signals are highlighted in blue, while HPPK–ligand complexes are highlighted in orange. (**B**) Chemical structure of 8-MG. (**C**) Titration curves of 8-MG at various concentrations against *M. smegmatis* HPPK (1 μM).

Prior to screening, the structural integrity and ionization behavior of recombinant *M. smegmatis* HPPK were evaluated under native MS conditions. The protein at 1 μM exhibited a narrow charge state distribution centered at 7+ and 8+, consistent with a compact, folded conformation (Figure 2A). The deconvoluted molecular mass of the apo-protein was 20,205 Da, in agreement with the theoretical mass calculated from the amino acid sequence, confirming the preservation of native-like structure during analysis.

8-Mercaptoguanine (8-MG, Figure 2B) is a previously reported ligand and inhibitor of bacterial HPPK enzymes, particularly against the *Staphylococcus aureus* HPPK (SaHPPK), where it was shown to bind within the pterin/substrate-binding pocket with a reported $K_{d}$ of approximately 11–13 μM and an IC_50_ of ~41 μM [60]. Structural and biophysical studies further demonstrated that the thiol group of 8-MG contributes significantly to binding affinity through interactions within the conserved substrate-binding region of HPPK [12]. In the present study, 8-MG was employed as a positive-control ligand to evaluate the binding competence of recombinant *M. smegmatis* HPPK under native MS conditions (Figure 2A). Direct observation of intact HPPK–8-MG complexes confirmed that the purified protein remained functionally folded and capable of ligand engagement during native MS analysis. Native MS titration experiments further indicated an apparent binding affinity in the low micromolar range, with an estimated $K_{d}$ of approximately 16.2 μM under the experimental conditions used (Figure 2C).

**Figure 3:**
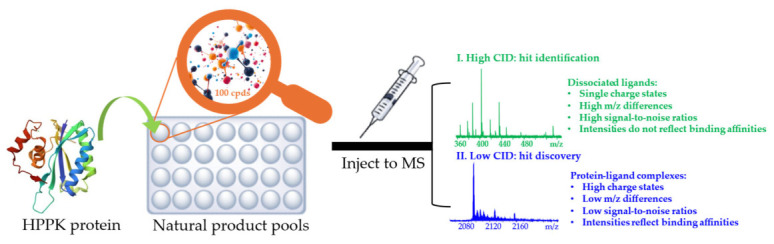
Native MS screening workflow. HPPK (10 μM) was incubated with natural product pools. Samples were analyzed under dual CID conditions: low CID (10 V) to preserve intact protein–ligand complexes and high CID (25–30 V) to induce ligand release. Hit identification was based on combined analysis of both datasets.

To enable high-throughput screening of complex mixtures, we developed a dual-mode native MS workflow for ligand detection and identification in pooled natural product libraries. In each experiment, HPPK (10 μM) was incubated with the compound pools (10 μM per compound; total ligand concentration 1 mM), followed by direct infusion into the mass spectrometer (Figure 3).

Native MS provides the sensitivity and resolution required to detect protein–ligand complexes in multiplexed mixtures. However, the low concentration of individual ligands results in weak complex signals, while the high total ligand load can induce ion suppression, further reducing signal intensity. In addition, high protein charge states compress *m*/*z* spacing between complexes, limiting confident assignment of bound species based solely on intact masses (e.g., a 5 Da mass difference at 10+ corresponds to only 0.5 *m*/*z* units).

To address these challenges, a dual-mode acquisition strategy combining low- and high-energy collision-induced dissociation (CID) was employed. Low CID preserves intact protein–ligand complexes for detection of binding events and estimation of relative binding affinities. The exact voltage for low CID represents a balance between maintaining complex integrity and maximizing protein signal intensity for detection. Based on method optimization with a positive control ligand, CID voltages in the range of 5–10 V did not disrupt the protein–ligand complex; 10 V was selected to provide a stronger protein signal while still preserving intact complexes. In contrast, high CID is expected to induce dissociation, releasing ligands as singly charged ions with broader *m*/*z* spacing and improved signal-to-noise ratios, enabling confident ligand identification. The high-CID range was determined using the positive control and prior studies [45,61], where voltages of 25–30 V resulted in efficient dissociation of bound ligands while maintaining the protein in a native-like state.

Importantly, the intensity of released ligand ions is influenced by ionization efficiency and therefore does not reliably reflect binding strength, whereas the intensity of intact protein–ligand complexes under low CID provides a more direct measure of apparent relative affinity under fixed screening conditions. By integrating both modes—complex detection for quantification and ligand release for identification—this workflow enables confident ligand assignment while retaining comparative information on intact complex formation. This approach is particularly well-suited for screening structurally diverse libraries, where binding is often weak and spectral complexity is high.

The relationship between intact complex abundance and apparent relative affinity is most informative when binding is not saturated and complex peaks are well resolved. According to our previous work, the natural-product ligands screened here showed weak binding responses, and 1:1 protein/ligand screening at 10 µM is therefore expected to remain sensitive to differences in binding strength.

### 2.2. Automated Data Analysis

**Figure 4:**
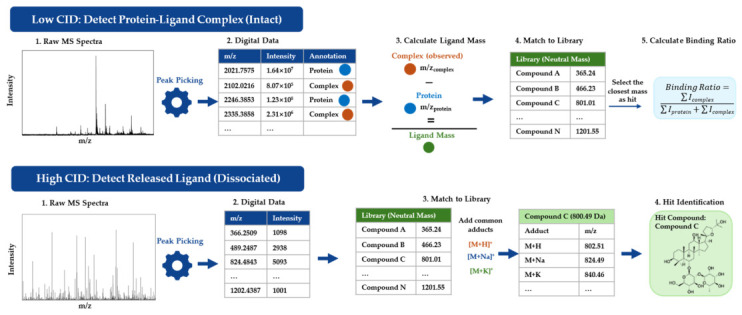
Python-based data analysis workflow for dual-mode native MS screening of ligand libraries.

To handle the high data volume and complexity of the 100-compound pooled screening experiments, we implemented a custom automated data analysis pipeline (Figure 4). This workflow was necessary to ensure consistent, high-throughput processing of the dual-mode native MS data across ~2000 compounds. Manual interpretation of such multiplexed spectra would be impractical and prone to error, especially given the weak signals and significant ion suppression observed in 100-ligand mixtures. By automating key steps of spectral processing and analysis, the pipeline systematically flags putative protein–ligand complexes in the low-collision energy (intact complex) datasets and assigns corresponding ligand identities using the high-collision energy (ligand-release) datasets, providing a robust foundation for hit identification.

Raw data from both low- and high-CID acquisitions were first processed using a peak-picking filter to extract centroided spectra, followed by conversion to mzML format using MSConvert for automated analysis. Centroided spectra from multiple scans were aligned to generate an apex spectrum for each sample.

For low-CID datasets, protein charge states were assigned based on the deconvoluted protein mass obtained from the protein-only spectrum, supported by the isotopic distribution of HPPK, and the two most intense charge states were selected for downstream analysis. Protein–ligand complexes were identified as newly emerged peaks adjacent to the apo-protein signals. Consistent mass differences observed across these charge states were used to define putative binding events. These mass differences were matched against the ligand database within a tolerance of ±2 Da, and each observed complex was assigned to a single ligand candidate based on the closest mass match to minimize ambiguity in highly multiplexed mixtures. Binding ratios were calculated as the relative intensity of the protein–ligand complex compared to the total protein signal (free and bound).

For high-CID datasets, protein–ligand complexes were dissociated, and released ligand ions were detected with reduced charge states and increased *m*/*z* separation. The molecular weights derived from the low-CID datasets were used to generate the expected adduct forms ([M + H]^+^, [M + Na]^+^, [M + K]^+^), and peaks were searched within the corresponding *m*/*z* regions in the high-CID spectra. The most intense peaks in these regions were selected and matched against the ligand database based on minimal mass difference.

Due to the limited resolution of intact protein–ligand complexes under native MS conditions, particularly for weak-binding ligands, mass differences derived from low-CID data may deviate from theoretical values. In contrast, released ligand ions detected under high-CID conditions provide improved mass accuracy and facilitate more confident matching to the ligand database. Integration of these complementary datasets enables reconciliation of complex-derived mass differences with directly observed ligand signals, thereby improving the reliability of ligand identification.

The workflow supports batch processing, automated binding ratio calculation, and visualization of results. Consistent detection of low-intensity complexes across dominant charge states was considered sufficient evidence for inclusion as true binding events.

### 2.3. Screening Results

Using the dual-mode native MS screening workflow in combination with automated data analysis, hit ligands from the screened natural product library were identified, using the binding ratio threshold of 1% in low-energy CID datasets. The binding threshold was low as the screening was done in an extremely complex pool of 100 compounds; ligand competition and signal suppression from high ligand concentrations could potentially influence the binding ratios.

A total of 14 ligands were detected to form protein–ligand complexes with HPPK (Figures S2 and S3, Table S1). Using a hit rate of 0.25%, the top five ligands were selected as initial hits (Table 1), all of which—pseudoginsenoside F11, ginsenoside Rb1, ginsenoside F3, ginsenoside Re, and ginsenoside Rb3—belong to the ginsenoside family. Among these, pseudoginsenoside F11 exhibited the highest complex intensity, with a binding ratio approaching 2%. The remaining four ginsenosides showed binding ratios ranging from approximately 1.0% to 1.5%.

However, in the 100-compound pooled screen, weak complex signals, ionization competition, and partial spectral overlap can affect apparent binding ratios. Therefore, binding ratios from the pooled screen were used primarily for hit detection and prioritization rather than definitive affinity ranking. Individual confirmation experiments, performed without other pool components with cleaner spectra need to be performed to compare relative binding strength among confirmed hits.

### 2.4. Hit Validation

**Figure 5:**
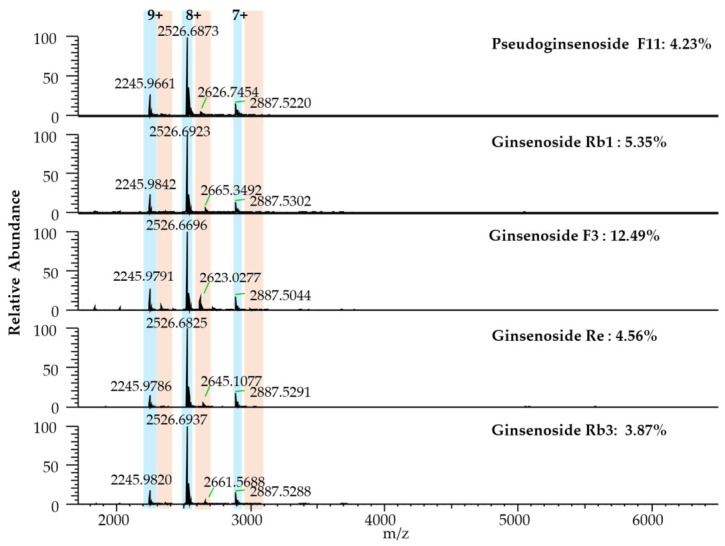
Native MS validation of individual binding of the five initial ginsenoside hits (25 μM) to HPPK (10 μM). The top panel shows HPPK alone, displaying the 7+, 8+, and 9+ charge states. The lower panels show spectra acquired in the presence of pseudoginsenoside F11, ginsenoside Rb1, ginsenoside F3, ginsenoside Re, and ginsenoside Rb3, respectively. Apo HPPK signals are highlighted in blue, while HPPK–ligand complexes are highlighted in orange.

**Figure 6:**
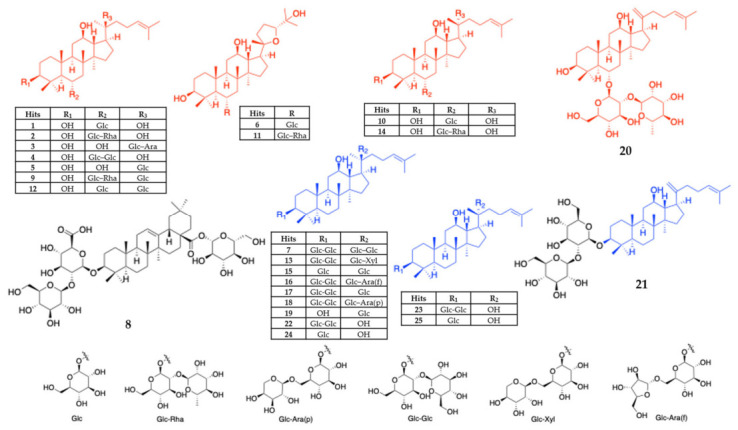
Structures of ginsenosides **1**–**25**. The protopanaxatriol (PPT) and protopanaxadiol (PPD) skeletons are highlighted in red and blue, respectively.

The identified hits were re-evaluated individually against HPPK at a protein–ligand ratio of 1:2.5 to validate their binding under non-competitive conditions (Figure 5). The resulting binding ratios are summarized in Figure 6 and Table 2. All five hits exhibited increased binding ratios in the individual measurements compared to the initial mixture screening.

Among them, ginsenoside F3 (**3**) showed the most pronounced enhancement, with more than a tenfold increase in binding ratio, reaching 12.49% under individual conditions. The remaining four compounds displayed binding in the order of ginsenoside Rb1 (**7**, 5.35%), ginsenoside Re (**9**, 4.56%), pseudoginsenoside F11 (**11**, 4.23%), and ginsenoside Rb3 (**13**, 3.87%).

In addition to the five initial hits identified from pooled screening, 20 additional ginsenosides were individually evaluated for HPPK binding at a protein-to-ligand ratio of 1:2.5 (Figure 4, Table 2 and Figure S4). Binding complexes were detected for the majority of compounds tested, while seven ginsenosides showed no detectable complex formation under the conditions used.

Among all individually tested compounds, the highest binding ratios were observed for (S)-ginsenoside Rh1 (**1**, 14.40%) and ginsenoside Rg2 (**2**, 13.66%), both of which exceeded that of ginsenoside F3 (**3**, 12.49%), the strongest ligand identified from the initial screening. Two additional ginsenosides, ginsenoside Rf (**4**, 11.35%) and ginsenoside F1 (**5**, 10.08%), also showed binding ratios above 10%. Notably, all of these highest-affinity ligands belong to the protopanaxatriol (PPT)-type subclass.

A clear structure–activity relationship was observed between the PPT- and protopanaxadiol (PPD)-type ginsenosides. The key structural difference between PPT- and PPD-type ginsenosides lies at the C-6 position. PPT-type ginsenosides possess a hydroxyl group at C-6, which is frequently substituted by glycosidic moieties, whereas PPD-type ginsenosides lack oxygenation at this position. Among the 18 ginsenosides for which binding was detected, 11 were PPT-type compounds, excluding ginsenoside Ro, which does not belong to the dammarane-type scaffold. More importantly, the strongest binders were exclusively PPT-type ginsenosides. By contrast, the PPD-type ginsenosides generally exhibited substantially weaker binding, and six of the seven non-binding ginsenosides belonged to this subclass.

These results indicate that the oxygenation at C-6 is an important structural determinant for HPPK recognition. This feature may enhance binding by providing an additional polar interaction site, either directly through the C-6 hydroxyl group or indirectly through a glycosidic substituent attached at this position.

The degree of glycosylation also appears to play an important role in modulating binding. The six strongest binders (**1**–**6**) all contain only one or two glycosidic substituents at the C-6 or C-20 positions, whereas more highly glycosylated ginsenosides, such as ginsenoside Rb1 (**7**, four sugars), ginsenoside Ro (**8**, three sugars), and ginsenoside Re (**9**, three sugars), exhibited reduced binding.

Increased glycosylation is expected to enhance overall polarity and steric bulk, which may limit the ability of the aglycone core to adopt a binding-competent conformation and reduce favorable interactions with the protein surface. Partially deglycosylated ginsenosides likely provide a more balanced presentation of polar and hydrophobic features, enabling more effective engagement with HPPK.

To further examine the role of physicochemical properties in binding, the cLogP values (calculated by DataWarrior v06.01.00) of all tested ginsenosides were analyzed. The strongest ligands (**1**–**6**) exhibited intermediate cLogP values ranging from 2.1 to 4.0. In contrast, weaker binders (**7**–**18**) generally showed lower cLogP values, with the exception of (R)-ginsenoside Rh1 (**10**) and (R)-ginsenoside Rg2 (**14**), which share identical cLogP values with their corresponding C-20 epimers. The lowest cLogP values were observed for Rb1 (**7**, −0.7), Ro (**8**, 0.2), Rb3 (**13**, 0.4), Rc (**16**, −0.2), and Rb2 (**18**, −0.1), all of which exhibited binding ratios below 6%.

Notably, most of the non-binding ginsenosides were predominantly associated with higher cLogP values, including ginsenoside Rg6 (**20**, 4.1), ginsenoside Rk1 (**21**, 4.0), and both (S)- and (R)-ginsenoside Rh2 (**24**–**25**, 4.8). These data suggest that an optimal range of hydrophobicity is required for productive interaction with HPPK. Compounds with low cLogP values are likely too polar, which may limit effective interaction with the protein surface, whereas highly hydrophobic ginsenosides may suffer from reduced solubility and/or an unfavourable presentation of polar functional groups required for binding.

A clear dependence on the C-20 configuration was also observed. Among the tested ginsenosides, there were four pairs of stereoisomers differing only at C-20, including two PPD-type pairs, (S/R)-ginsenoside Rg3 (**22** and **23**) and (S/R)-ginsenoside Rh2 (**24** and **25**), which did not exhibit detectable binding to HPPK in either configuration. For the two PPT-type pairs, (S)-ginsenoside Rh1 (**1**, 14.40%) and (S)-ginsenoside Rg2 (**2**, 13.66%) showed substantially stronger binding than their corresponding 20(R) counterparts, (R)-ginsenoside Rh1 (**10**, 4.44%) and (R)-ginsenoside Rg2 (**14**, 3.62%), corresponding to 3.1-fold and 3.6-fold differences, respectively.

The orientation at C-20 alters the spatial arrangement and conformational flexibility of the side chain, thereby modulating the three-dimensional presentation of the C-20 substituent and neighboring functional groups. This, in turn, can significantly influence the positioning of glycosidic moieties and polar functionalities relative to the protein surface. Previous studies have demonstrated that 20(S) and 20(R) epimers of ginsenosides often exhibit markedly different biological activities across a range of systems, including modulating lipid mediator profiles [62], antioxidant, anti-cancer [63], anti-inflammatory [64], and regenerative effects [65], consistent with differences in their ability to engage in productive molecular interactions.

## 3. Discussion

In this study, native MS was applied to screen a natural product library for ligands binding to HPPK. A dual-mode acquisition strategy was implemented, combining low-CID conditions for detection of intact protein–ligand complexes with high-CID conditions for ligand release and identification. This design enables complementary readouts from a single experiment, allowing both binding detection and ligand assignment in highly multiplexed samples. In addition, the development of an automated Python-based data analysis workflow ensures consistent processing of complex datasets and supports scalable application of this approach to larger libraries and higher-throughput screening campaigns.

Screening results revealed a clear enrichment of ginsenoside-related compounds among the detected binders. Analysis of this compound class showed a consistent structure–binding relationship, where binding strength is influenced by scaffold type, glycosylation pattern, and stereochemistry. In particular, PPT-type ginsenosides exhibited stronger binding compared to PPD-type analogues, and ligands with limited and strategically positioned glycosylation showed more favourable interactions. Small variations in sugar substitution may alter ligand orientation or introduce steric effects that affect how the molecule interacts with the protein surface, leading to distinct binding behaviors among closely related structures [66,67]. These observations indicate that HPPK recognition is sensitive to both the physicochemical properties and three-dimensional presentation of ginsenoside ligands, highlighting this class as a structurally informative set for probing protein–ligand interactions.

Although the identified ginsenosides exhibited clear and structure-dependent binding to HPPK, none of the tested compounds showed significant inhibition of *M. smegmatis* growth at concentrations up to 100 μM. Similar discrepancies between enzyme inhibition and antibacterial activity have also been reported for previously described high-affinity HPPK inhibitors, which show strong inhibition in biochemical assays but limited antibacterial effects in bacterial systems [68]. The high intracellular concentration of ATP and the highly optimized catalytic mechanism of the enzyme may limit the effectiveness of small-molecule inhibitors [69,70]. For an ATP-dependent enzyme such as HPPK, functional inhibition would require a ligand to bind with sufficient affinity and orientation to interfere with ATP- or substrate-dependent catalysis. In addition, cellular activity depends on compound uptake, retention, and stability within mycobacterial cells, which may be challenging for highly glycosylated ginsenosides.

The ability of these detected ginsenosides to bind HPPK in a structure-dependent manner suggests that they engage defined regions of the protein surface, even if these interactions do not directly interfere with catalysis. This may reflect the challenge of targeting ATP-dependent enzymes, where functional inhibition requires precise engagement of the catalytic machinery. In this context, ginsenosides provide a useful starting point for further investigation of ligand recognition, and may serve as a basis for future optimization toward functionally active compounds. More broadly, the results demonstrate that native MS-based screening can uncover structurally coherent ligand classes and provide insight into molecular recognition processes.

Future studies incorporating orthogonal biophysical methods such as surface plasmon resonance (SPR) and isothermal titration calorimetry (ITC) will be valuable for defining the binding affinities and binding modes of the identified ginsenosides in greater detail. Such studies will further clarify the molecular basis underlying the observed structure-dependent binding behaviour and support the development of optimized ligands targeting HPPK.

## 4. Materials and Methods

### 4.1. Ligand Preparation

8-Mercaptoguanine (8-MG) was purchased from Combi-Blocks, Inc. (San Diego, CA, USA).

The Quinn−Liu natural product library (~1890 compounds) was plated by Compounds Australia (Griffith University, QLD, Australia) into 19 pools of up to 100 compounds each (with any pools containing fewer than 100 compounds supplemented with equivalent volumes of DMSO to maintain consistent total volume and concentration). All compounds in each pool were present at equal nominal concentration (50 µM per compound in 100% DMSO stocks).

### 4.2. Protein Expression and Purification

Recombinant 2-amino-4-hydroxy-6-hydroxymethyldihydropteridine pyrophosphokinase (Rv3606c ortholog, HPPK) from *Mycobacterium smegmatis* was supplied as a purified protein by the Seattle Structural Genomics Center for Infectious Diseases (SSGCID, Seattle, WA, USA; protein ID MysmA.01570.a.B1.PS02122). The protein corresponded to full-length *M. smegmatis* HPPK/FolK residues 1–178 and contained an N-terminal His-tag sequence (MAHHHHHH) preceding the native sequence. The recombinant protein was expressed in *E. coli* BL21(DE3) Rosetta/BL21(DE3)R3 Rosetta using auto-induction medium and purified by His-tag-based Ni^2+^ affinity chromatography followed by size-exclusion chromatography. The protein was stored under −80 °C in a buffer containing 20 mM HEPES (pH 7.0), 300 mM NaCl, 5% (*v*/*v*) glycerol, and 1 mM tris(2-carboxyethyl)phosphine (TCEP).

### 4.3. Sample Preparation for Native MS

The protein was buffer-exchanged into 300 mM ammonium acetate (pH ~ 7.5, Sigma-Aldrich, St. Louis, MO, USA) using an Amersham NAP-5 desalting column (Cytiva, Marlborough, MA, USA) to remove non-volatile salts and prepare the protein for native MS. The protein concentration in all native MS binding experiments was 10 µM.

To prepare screening samples for native MS, a 10 µL aliquot of the DMSO stock solution from a 100-compound pool was dried under vacuum using a freeze dryer to remove DMSO. The dried compound mixture was reconstituted in 1 µL of methanol with careful mixing and visual for any obvious undissolved material. The sample was then incubated with 49 µL of HPPK solution for 1 h. This resulted in a final screening mixture containing HPPK at 10 µM and each individual compound at approximately 10 µM, with a total ligand concentration of approximately 1 mM and 2% methanol.

For individual ligand validation experiments, selected hit compounds (along with additional related ginsenosides) were obtained as 500 µM DMSO stock solutions from Compounds Australia, Griffith University (Nathan, QLD, Australia) in 96-well plates. An aliquot of each compound (2.5 µL) was similarly dried, reconstituted in 1 µL of methanol, and incubated with 49 µL of 10 µM HPPK solution for 1 h prior to native MS analysis. All experiments were performed in duplicate.

### 4.4. Mass Spectrometry Instrument Control and Data Acquisition

Screening measurements were performed on a Bruker SolariX XR 12T FT-ICR mass spectrometer (Bruker Daltonics, Bremen, Germany). Samples were directly infused using a 500 μL Hamilton syringe mounted on the integrated syringe pump at a flow rate of 120 μL/h. The capillary voltage was set to 3500 V, with an end-plate offset of −800 V. Drying gas was delivered at a flow rate of 4 L/min, with a nebulizer pressure of 3 bar and a temperature of 200 °C. Source optics voltages were configured as follows: capillary exit 200 V, deflector plate 220 V, funnel 1 at 150 V, and skimmer 1 at 30 V. The optical transfer frequency was set to 4 MHz with a time-of-flight (TOF) of 1.0 ms. Collision-induced dissociation (CID) voltages of 10 V (low-CID) and 25–30 V (high-CID) were applied in separate acquisitions for each sample. Each mass spectrum was obtained by summing 16 transients, with each transient consisting of 1 million data points. Mass spectra were recorded in positive ion mode over an *m*/*z* range of 100–6000. Instrument control and data acquisition were performed using Bruker SolariX control software (ftmsControl), version 2.0 (Bruker Daltonics, Bremen, Germany).

Ligand validation and additional ginsenosides were analysed on a Q Exactive UHMR mass spectrometer (Thermo Fisher Scientific, Waltham, MA, USA) equipped with a Nanospray Flex ion source. Typical parameters were as follows: spray voltage 1.8 kV, capillary temperature 220 °C, S-lens RF level 200, AGC target fixed, maximum injection time 200 ms, and in-source trapping enabled. Ion optics were configured with a source DC offset of 21 V, desolvation voltage of 0 V, injection flatapole DC of 5 V, inter-flatapole lens of 4 V, bent flatapole DC of 2 V, transfer multipole DC of 0 V, and a C-trap entrance lens inject voltage of 1.8 V. RF amplitudes were set to 150 V for the injection flatapole, 300 V for the bent flatapole, 250 V for the transfer multipole and HCD cell, and 2300 V for the C-trap. The trapping gas pressure was set to 3.0 (arbitrary units), and the C-trap charge detector support was disabled. Data were typically acquired with 5 microscans at a scan rate of approximately 0.8 scans/s. Raw data were processed using FreeStyle software, version 1.8 (Thermo Fisher Scientific, USA).

### 4.5. Data Analysis

Mass spectrometry screening data from Bruker FTMS files were processed using a custom automated pipeline implemented in Python version 3.14 (Code S1).

First, data files from each acquisition mode (low-CID and high-CID/HCD) were centroided and converted to an open format (mzML) using the MSConvert tool (ProteoWizard). For each sample and each collision condition, multiple individual scans were aligned and combined (averaged) to generate a single representative “apex” spectrum to improve detection of low-intensity signals. Peak assignment and hit identification were then carried out by comparing the low-CID and high-CID spectra.

In the low-CID (intact complex) spectra, the charge states of the apo-HPPK ion signals were identified using the known mass (20,205 Da) and isotopic distribution of HPPK (as determined from a separate protein-only spectrum). The two most intense HPPK charge states were used for analysis of binding. New peaks corresponding to protein–ligand complex ions were detected as reproducible signals at higher *m*/*z* than the apo-HPPK peaks. The mass differences between each putative complex and the apo-protein (for each charge state) were calculated and compared against the molecular weights of compounds in the screened ligand pool. A mass tolerance of ±2 Da was used for matching experimental mass differences to entries in the ligand library database. Only those mass shifts that appeared consistently across at least two independent charge states were considered evidence of a binding event.

In parallel, the high-CID (ligand-release) spectra were analyzed to confirm the identities of the bound ligands: the expected *m*/*z* values of potential ligand adduct ions ([M + H]^+^, [M + Na]^+^, and [M + K]^+^ species) were calculated for each candidate hit from the low-CID analysis, and the high-CID spectra were searched for corresponding peaks. Detection of a matching ligand ion in the high-CID data, within the defined mass tolerance, was used to verify the assignment of each protein-bound mass shift to a specific ligand.

Binding ratios for HPPK–ligand complexes were computed from the low-CID spectra as the intensity of the ligand-bound protein peak divided by the total intensity of protein (free HPPK plus HPPK bound to that ligand) for the chosen charge state(s).

### 4.6. Titration of 8-MG Against HPPK by Native MS

8-MG was prepared at ten concentrations ranging from 1 to 80 μM in 1 μL methanol and incubated with 49 μL of HPPK solution for 1 h at room temperature prior to native MS analysis. All experiments were performed in triplicate.

Deconvolution of native MS spectra acquired from all instruments was carried out using UniDec (University of Oxford, UK). Charge-state-resolved intensities of apo and ligand-bound protein were then extracted from deconvoluted spectra.

The dissociation constant ($K_{d}$) values, defined as

$$K_{d}=\frac{\left[P\right]\times [L]}{\left[P-L\right]}=\frac{\left({\left[P\right]}_{0}-\left[P-L\right])\times ({\left[L\right]}_{0}-\left[P-L\right]\right)}{\left[P-L\right]}$$

where $\left[P\right]$, [L], ${\left[P\right]}_{0}$, ${\left[L\right]}_{0}$ and $\left[P-L\right]$ represent the concentrations of the free protein, free ligand, total protein, total ligand and protein–ligand complex, respectively.

The ratio of protein–ligand complex ($\left[P-L\right]$) was determined using the following equation:

$$\frac{\left[P-L\right]}{{\left[P\right]}_{0}}=\;\frac{\sum_{n}I_{{\left[P-L\right]}^{n+}}}{\sum_{n}\left(I_{{\left[P\right]}^{n+}}{+I}_{{\left[P-L\right]}^{n+}}\right)}$$

where $I_{{\left[P-L\right]}^{n+}}$ is the intensity of the $\left[P-L\right]$ at charge state *n*, $I_{{\left[P\right]}^{n+}}$ is the intensity of the apo-protein at charge state *n*, and the summation accounts for all observed charge states of the protein.

A binding curve was then generated by plotting ligand concentration (${\left[L\right]}_{0}$) against the percentage of $\left[P-L\right]$. Non-linear regression was performed in GraphPad Prism version 11 using the One-site-Specific binding model to obtain the fitted $K_{d}$ values.

## Figures and Tables

**Table 1:** Top five initial hits identified from native MS screening of HPPK.

Initial Hits	Expected MW (Da)	Calculated MW (Da) Under Low-CID Conditions	Detected Adduct Ions Under High-CID Conditions			Binding Ratios (%)
			[M + H]^+^	[M + Na]^+^	[M + K]^+^	
Pseudoginsenoside F11	801.01	801.43	802.5024	824.4843 *	840.4576	1.89
Ginsenoside Rb1	1109.29	1108.55	1109.6089	1131.5910 *	1147.5865	1.36
Ginsenoside F3	770.99	770.39	771.4890 *	793.4710	809.4448	1.12
Ginsenoside Re	947.15	947.09	-	970.5422 *	986.5373	1.09
Ginsenoside Rb3	1079.27	1079.93	1079.5979	1101.5804 *	1117.5546	1.02

* The most intense peak selected for downstream analysis.

**Table 2:** Structural features of the 25 ginsenosides tested for HPPK binding.

Hits	Name	Class	C-3	C-6	C-20	Number of Sugars	cLogP	Binding Ratios (%)
**1**	S-ginsenoside Rh1	PPT	OH	Glc	OH	1	4.0	14.40 ± 0.11
**2**	Ginsenoside Rg2	PPT	OH	Glc–Rha	OH	2	3.0	13.66 ± 0.69
**3**	Ginsenoside F3	PPT	OH	OH	Glc–Ara(p)	2	2.7	12.49 ± 0.17
**4**	Ginsenoside Rf	PPT	OH	Glc–Glc	OH	2	2.1	11.35 ± 0.54
**5**	Ginsenoside F1	PPT	OH	OH	Glc	1	4.0	10.08 ± 1.41
**6**	Pseudoginsenoside RT5	PPT	OH	Glc	20,24-epoxy	1	2.3	8.32 ± 0.49
**7**	Ginsenoside Rb1	PPD	Glc-Glc	H	Glc–Glc	4	−0.7	5.35 ± 0.03
**8**	Ginsenoside Ro	*	GlcA-Glc	H	H	3	0.2	5.09 ± 0.06
**9**	Ginsenoside Re	PPT	OH	Glc–Rha	Glc	3	1.2	4.56 ± 0.33
**10**	R-ginsenoside Rh1	PPT	OH	Glc	OH	1	4.0	4.44 ± 0.18
**1** **1**	Pseudoginsenoside F11	PPT	OH	Glc–Rha	20,24-epoxy	2	1.4	4.23 ± 0.19
**12**	Ginsenoside Rg1	PPT	OH	Glc	Glc	2	2.1	3.98 ± 0.25
**13**	Ginsenoside Rb3	PPD	Glc-Glc	H	Glc–Xyl	4	0.4	3.87 ± 0.22
**14**	R-ginsenoside Rg2	PPT	OH	Glc–Rha	OH	2	3.0	3.62 ± 0.08
**15**	Ginsenoside F2	PPD	Glc	H	Glc	2	3.0	3.60 ± 0.08
**16**	Ginsenoside Rc	PPD	Glc-Glc	H	Glc–Ara(f)	4	−0.2	3.46 ± 0.09
**17**	Ginsenoside Rd	PPD	Glc-Glc	H	Glc	3	1.1	2.65 ± 0.18
**18**	Ginsenoside Rb2	PPD	Glc-Glc	H	Glc–Ara(p)	4	−0.1	2.29 ± 0.04
**19**	Ginsenoside CK	PPD	OH	H	Glc	1	4.8	0
**20**	Ginsenoside Rg6	PPT	OH	Glc–Rha	#	2	4.1	0
**21**	Ginsenoside Rk1	PPD	Glc-Glc	H	#	2	4.0	0
**22**	S-ginsenoside Rg3	PPD	Glc-Glc	H	OH	2	3.0	0
**23**	R-ginsenoside Rg3	PPD	Glc-Glc	H	OH	2	3.0	0
**24**	S-ginsenoside Rh2	PPD	Glc	H	OH	1	4.8	0
**25**	R-ginsenoside Rh2	PPD	Glc	H	OH	1	4.8	0

* Ginsenoside Ro: oleanane-type (oleanolic acid-based), not PPD/PPT. # Dehydrated side chain.

## Data Availability

Dataset available on request from the corresponding author.

## Supplementary Materials

The following supporting information can be downloaded at https://www.mdpi.com/article/10.3390/molecules31122065/s1, Figure S1: Pairwise sequence alignment and conserved motif annotation of HPPK from *Mycobacterium tuberculosis* and *M. smegmatis*; Figure S2: Native MS screening spectra for HPPK-binding ligands acquired under low-CID conditions (10 V); Figure S3: Native MS screening spectra for HPPK-binding ligands acquired under high-CID conditions (25–30 V); Figure S4: Native MS spectra of 20 additional ginsenosides (25 μM) against HPPK (10 μM); Table S1: Automated data analysis results from dual-mode native MS pooled screening of HPPK against 100-compound natural product pools under low-CID and high-CID conditions; Code S1: Python script for automated analysis of integrated dual-mode native MS datasets.

## Abbreviations

The following abbreviations are used in this manuscript:

HPPK	6-hydroxymethyl-7,8-dihydropterin pyrophosphokinase
TB	Tuberculosis
*Mtb*	*Mycobacterium tuberculosis*
MDR-TB	multidrug-resistant TB
MS	mass spectrometry
CID	collision-induced dissociation
PPT	protopanaxatriol
PPD	protopanaxadiol
SSGCID	Seattle Structural Genomics Center for Infectious Diseases
TOF	time-of-flight
