# Supplementary material for: Dual-Mode Native Mass Spectrometry Screening Identifies Ginsenoside Ligands of 6-Hydroxymethyl-7,8-Dihydropterin Pyrophosphokinase (HPPK)
Source: Molecules. 2026 Jun 12;31(12):2065. doi: 10.3390/molecules31122065 (PMC13304546; doi:10.3390/molecules31122065)
Supplement: Supplementary file 1 [file molecules-31-02065-s001.zip › molecules-4321180-supplementary.pdf]

# Supporting Information: Dual-Mode Native Mass Spectrometry Screening Identifies Ginsenoside Ligands of 6-hydroxymethyl-7,8-dihydropterin pyrophosphokinase (HPPK)

Xinru Xue[1], Ronald J Quinn[1], Bernd H.A. Rehm[1,2], Peter J. Myler [3], Miaomiao Liu[1,4,*]

[1] Institute for Biomedicine and Glycomics, Griffith University, Southport 4215, Gold Coast, Queensland, Australia;xinru.xue@griffithuni.edu.au(X.X.)

[2] Centre for Cell Factories and Biopolymers, Institute for Biomedicine and Glycomics, Griffith University, Southport 4215, Gold Coast, Queensland, Australia

[3] Center for Global Infectious Disease Research, Seattle Children's Research Institute, Seattle, WA 98109, USA

[4] School of Environment and Science, Griffith University, Nathan 4111, Brisbane, Queensland, Australia

*Correspondence: miaomiao.liu@griffith.edu.au; Tel.: +61-7-3735-4412

Table of Contents

Figure S1. Pairwise sequence alignment and conserved motif annotation of HPPK from *Mycobacterium tuberculosis* and *M. smegmatis*.

Figure S2. Native MS screening spectra for HPPK-binding ligands acquired under low-CID conditions (10 V).

Figure S3. Native MS screening spectra for HPPK-binding ligands acquired under high-CID conditions (25-30 V).

Figure S4. Native MS spectra of 20 additional ginsenosides (25 μM) against HPPK (10 μM).

Table S1. Automated data analysis results from dual-mode native MS pooled screening of HPPK against 100-compound natural product pools under low-CID and high-CID conditions.

Code S1. Python script for automated analysis of integrated dual-mode native MS datasets.

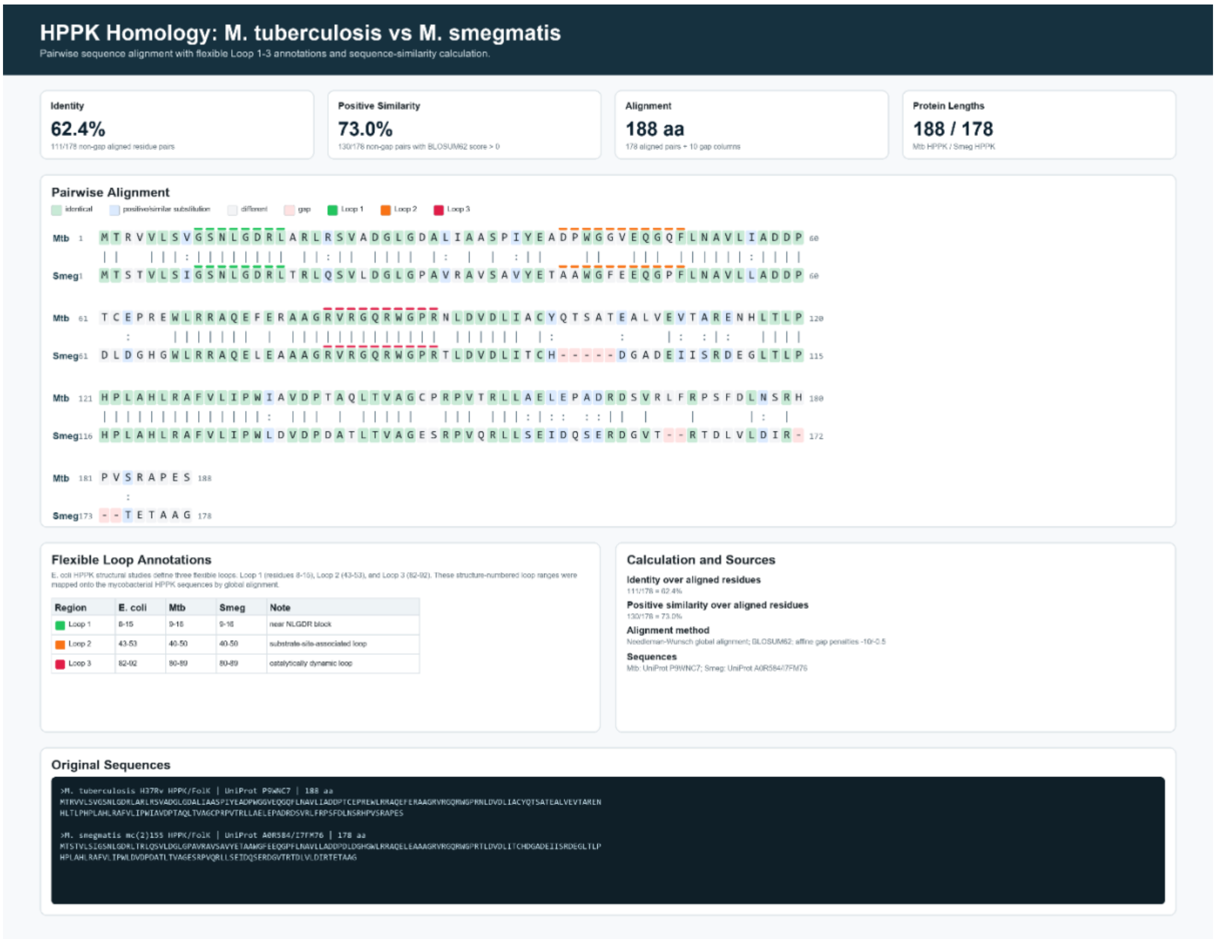


**Figure S1. Pairwise sequence alignment and conserved motif annotation of HPPK from *Mycobacterium tuberculosis* and *M. smegmatis*.**

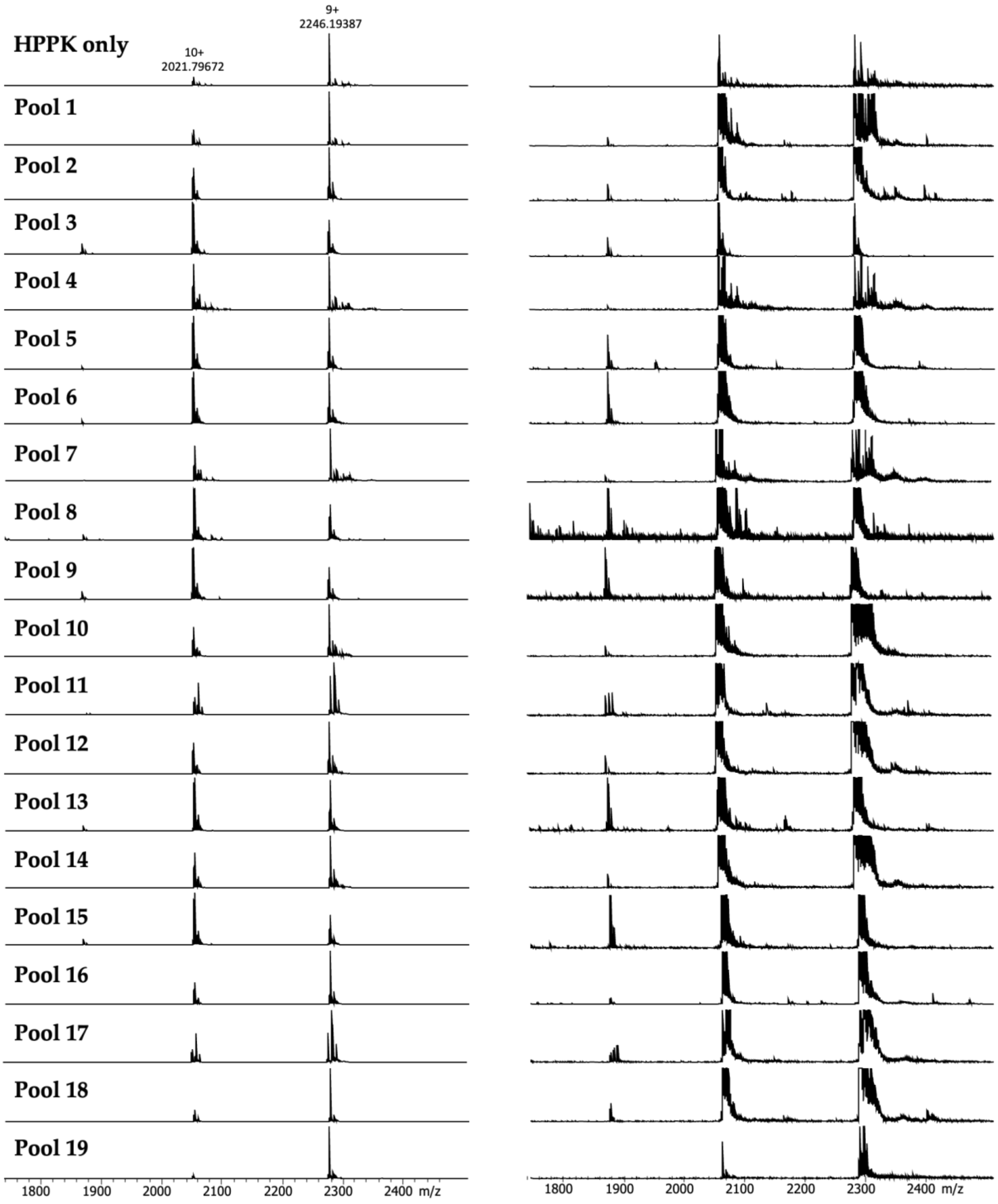


**Figure S2. Native MS screening spectra for HPPK-binding ligands acquired under low-CID conditions (10 V).** From top to bottom: HPPK-only control, followed by HPPK incubated with 19 ligand pools (100 compounds per pool; Pools 1–19). Expanded spectra are shown on the right.

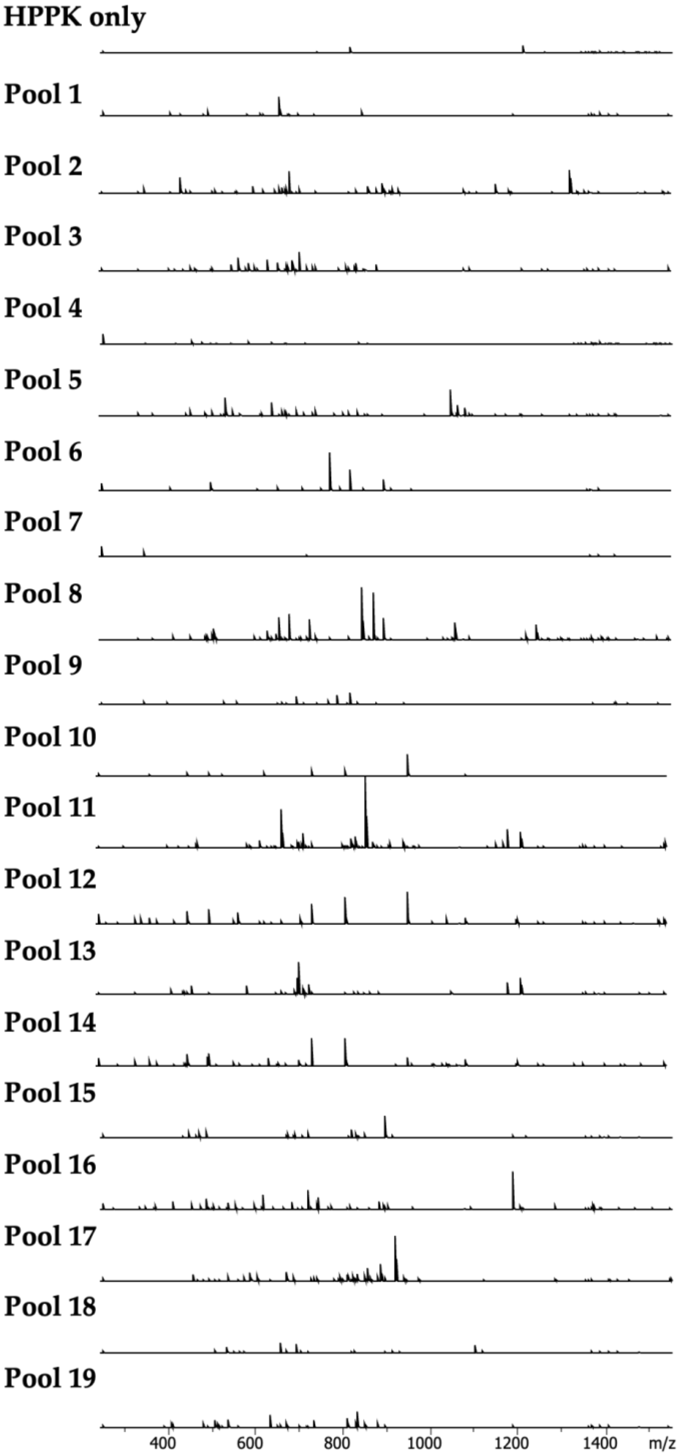


**Figure S3. Native MS screening spectra for HPPK-binding ligands acquired under high-CID conditions (25-30 V).** From top to bottom: HPPK-only control, followed by HPPK incubated with 19 ligand pools (100 compounds per pool; Pools 1–19).

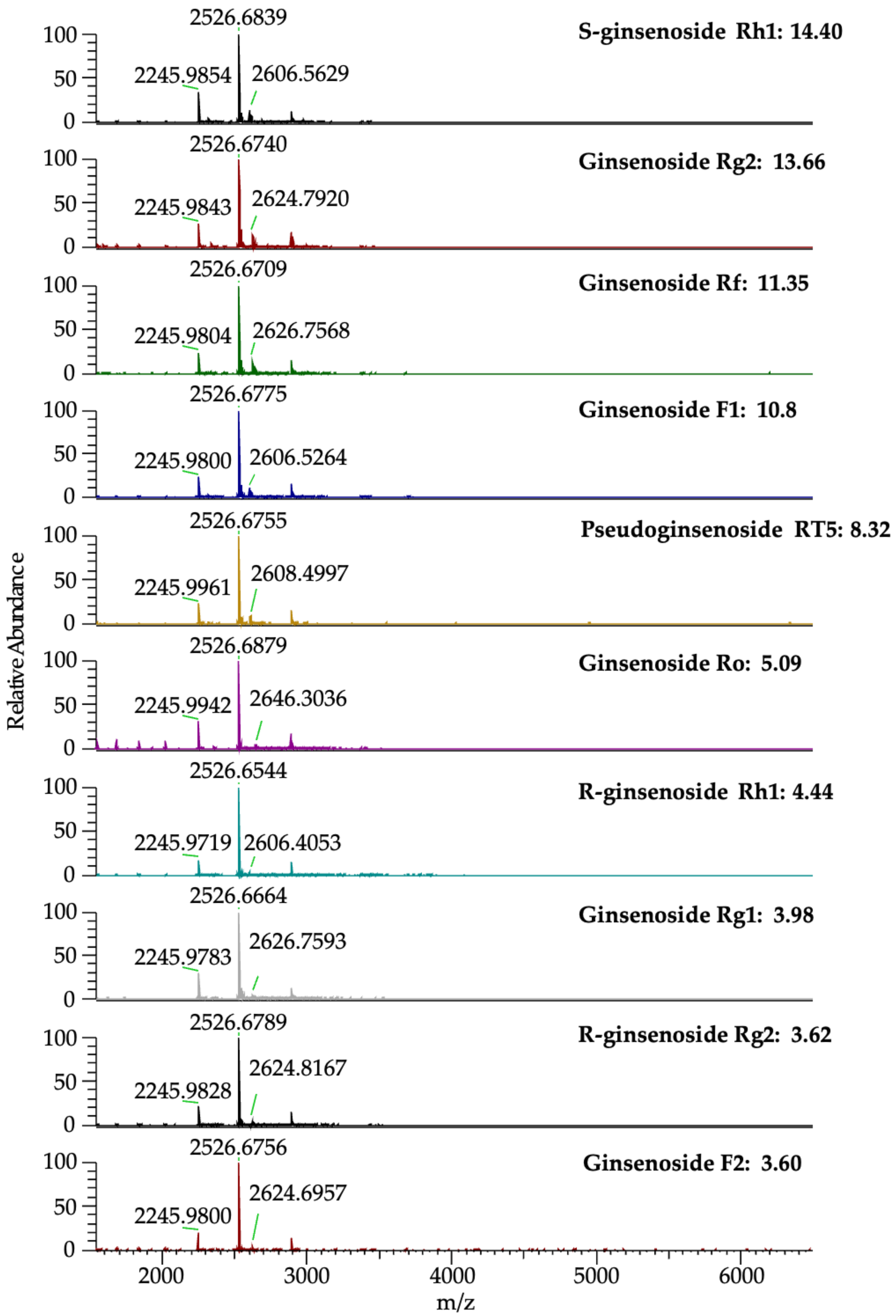


**Figure S4. Native MS spectra of 20 additional ginsenosides (25 μM) against HPPK (10 μM).**

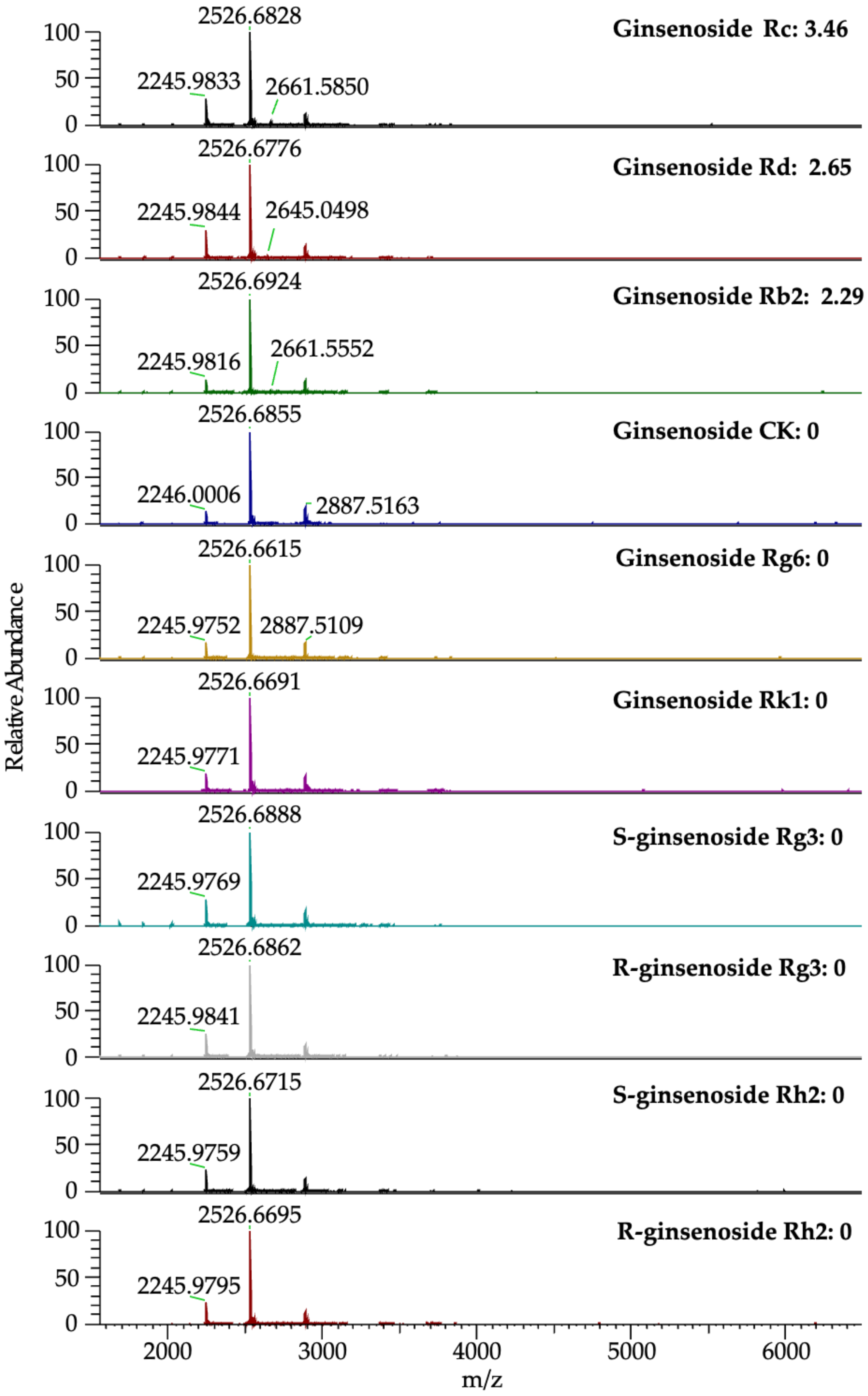


Figure S4. Continued.

**Table S1. Automated data analysis results from dual-mode native MS pooled screening of HPPK against 100-compound natural product pools under low-CID and high-CID conditions.**

<table>
<tr><th rowspan="3">Pool</th><th colspan="8">Low-CID MS</th><th colspan="5">High-CID MS</th><th rowspan="3">Nearest ligand mass</th></tr>
<tr><th rowspan="2">Charge state</th><th colspan="2">Apo-protein (P)</th><th colspan="2">Complex (P-L)</th><th rowspan="2">Ligand mass</th><th rowspan="2">Ratio (P-L/P)</th><th rowspan="2">Binding ratio (%)</th><th rowspan="2">Adducts</th><th rowspan="2">Theoretical</th><th rowspan="2">Observed</th><th rowspan="2">Δm/z</th><th rowspan="2">Intensity</th></tr>
<tr><th>m/z</th><th>Intensity</th><th>m/z</th><th>Intensity</th></tr>

<tr><td rowspan="6">8</td><td rowspan="3">9+</td><td rowspan="3">2246.38532</td><td rowspan="3">1.23E+08</td><td rowspan="3">2335.38578</td><td rowspan="3">2.31E+06</td><td rowspan="3">801.00411</td><td rowspan="3">0.018826</td><td rowspan="6">1.89</td><td rowspan="2">[M+H]+</td><td rowspan="2">802.50241</td><td rowspan="2">802.50241</td><td rowspan="2">0</td><td rowspan="2">1.58E+07</td><td rowspan="6">801.01</td></tr>
<tr></tr>
<tr><td rowspan="2">[M+Na]+</td><td rowspan="2">824.48435</td><td rowspan="2">824.48434</td><td rowspan="2">-1.37E-05</td><td rowspan="2">7.49E+06</td></tr>
<tr><td rowspan="3">10+</td><td rowspan="3">2021.75747</td><td rowspan="3">1.64E+07</td><td rowspan="3">2101.87606</td><td rowspan="3">3.71E+05</td><td rowspan="3">801.18595</td><td rowspan="3">0.022594</td></tr>
<tr><td rowspan="2">[M+K]+</td><td rowspan="2">840.45829</td><td rowspan="2">840.45759</td><td rowspan="2">-0.00071</td><td rowspan="2">6.11E+05</td></tr>
<tr></tr>

<tr><td rowspan="6">13</td><td rowspan="3">9+</td><td rowspan="3">2246.06487</td><td rowspan="3">3.93E+08</td><td rowspan="3">2369.23912</td><td rowspan="3">3.68E+06</td><td rowspan="3">1108.56823</td><td rowspan="3">0.009379</td><td rowspan="6">1.36</td><td rowspan="2">[M+H]+</td><td rowspan="2">1109.60917</td><td rowspan="2">1109.60887</td><td rowspan="2">-0.0003</td><td rowspan="2">6.41E+05</td><td rowspan="6">1109.29</td></tr>
<tr></tr>
<tr><td rowspan="2">[M+Na]+</td><td rowspan="2">1131.59111</td><td rowspan="2">1131.59111</td><td rowspan="2">0</td><td rowspan="2">1.32E+07</td></tr>
<tr><td rowspan="3">10+</td><td rowspan="3">2021.45945</td><td rowspan="3">4.11E+08</td><td rowspan="3">2132.31283</td><td rowspan="3">7.40E+06</td><td rowspan="3">1108.53379</td><td rowspan="3">0.017974</td></tr>
<tr><td rowspan="2">[M+K]+</td><td rowspan="2">1147.56505</td><td rowspan="2">1147.58654</td><td rowspan="2">0.02149</td><td rowspan="2">9.23E+05</td></tr>
<tr></tr>

<tr><td rowspan="6">11</td><td rowspan="3">9+</td><td rowspan="3">2246.21915</td><td rowspan="3">3.97E+08</td><td rowspan="3">2331.87577</td><td rowspan="3">3.96E+06</td><td rowspan="3">770.90957</td><td rowspan="3">0.009983</td><td rowspan="6">1.12</td><td rowspan="2">[M+H]+</td><td rowspan="2">772.49248</td><td rowspan="2">772.49253</td><td rowspan="2">5.69E-05</td><td rowspan="2">4.47E+06</td><td rowspan="6">770.99</td></tr>
<tr></tr>
<tr><td rowspan="2">[M+Na]+</td><td rowspan="2">794.47442</td><td rowspan="2">794.47442</td><td rowspan="2">0</td><td rowspan="2">2.68E+07</td></tr>
<tr><td rowspan="3">10+</td><td rowspan="3">2021.59403</td><td rowspan="3">1.80E+08</td><td rowspan="3">2098.75266</td><td rowspan="3">2.59E+06</td><td rowspan="3">771.58631</td><td rowspan="3">0.014381</td></tr>
<tr><td rowspan="2">[M+K]+</td><td rowspan="2">810.44836</td><td rowspan="2">810.4481</td><td rowspan="2">-0.00025</td><td rowspan="2">2.29E+06</td></tr>
<tr></tr>

<tr><td rowspan="6">5</td><td rowspan="3">9+</td><td rowspan="3">2246.06487</td><td rowspan="3">5.70E+08</td><td rowspan="3">2351.24745</td><td rowspan="3">7.45E+06</td><td rowspan="3">946.64323</td><td rowspan="3">0.013074</td><td rowspan="6">1.09</td><td rowspan="2">[M+H]+</td><td rowspan="2">948.56028</td><td rowspan="2">-</td><td rowspan="2">-</td><td rowspan="2">-</td><td rowspan="6">947.15</td></tr>
<tr></tr>
<tr><td rowspan="2">[M+Na]+</td><td rowspan="2">970.54222</td><td rowspan="2">970.54222</td><td rowspan="2">0</td><td rowspan="2">1.07E+07</td></tr>
<tr><td rowspan="3">10+</td><td rowspan="3">2021.55557</td><td rowspan="3">5.91E+08</td><td rowspan="3">2116.32637</td><td rowspan="3">5.35E+06</td><td rowspan="3">947.70797</td><td rowspan="3">0.009045</td></tr>
<tr><td rowspan="2">[M+K]+</td><td rowspan="2">986.51616</td><td rowspan="2">986.53726</td><td rowspan="2">0.02109</td><td rowspan="2">4.27E+06</td></tr>
<tr></tr>

<tr><td rowspan="6">13</td><td rowspan="3">9+</td><td rowspan="3">2246.06487</td><td rowspan="3">3.93E+08</td><td rowspan="3">2365.90316</td><td rowspan="3">2.80E+06</td><td rowspan="3">1078.54465</td><td rowspan="3">0.007132</td><td rowspan="6">1.02</td><td rowspan="2">[M+H]+</td><td rowspan="2">1079.5985</td><td rowspan="2">1079.59789</td><td rowspan="2">-0.00061</td><td rowspan="2">3.09E+05</td><td rowspan="6">1079.27</td></tr>
<tr></tr>
<tr><td rowspan="2">[M+Na]+</td><td rowspan="2">1101.58044</td><td rowspan="2">1101.58044</td><td rowspan="2">0</td><td rowspan="2">9.95E+06</td></tr>
<tr><td rowspan="3">10+</td><td rowspan="3">2021.45945</td><td rowspan="3">4.11E+08</td><td rowspan="3">2129.41832</td><td rowspan="3">5.45E+06</td><td rowspan="3">1079.5887</td><td rowspan="3">0.013256</td></tr>
<tr><td rowspan="2">[M+K]+</td><td rowspan="2">1117.55438</td><td rowspan="2">1117.55456</td><td rowspan="2">0.00018</td><td rowspan="2">5.44E+05</td></tr>
<tr></tr>

<tr><td rowspan="6">2</td><td rowspan="3">9+</td><td rowspan="3">2246.17168</td><td rowspan="3">1.07E+09</td><td rowspan="3">2362.26151</td><td rowspan="3">1.11E+07</td><td rowspan="3">1044.80852</td><td rowspan="3">0.010418</td><td rowspan="6">0.93</td><td rowspan="2">[M+H]+</td><td rowspan="2">1045.55672</td><td rowspan="2">1045.55735</td><td rowspan="2">0.00063</td><td rowspan="2">7.30E+05</td><td rowspan="6">1044.6</td></tr>
<tr></tr>
<tr><td rowspan="2">[M+Na]+</td><td rowspan="2">1067.53866</td><td rowspan="2">1067.53866</td><td rowspan="2">0</td><td rowspan="2">7.83E+06</td></tr>
<tr><td rowspan="3">10+</td><td rowspan="3">2021.60364</td><td rowspan="3">6.52E+08</td><td rowspan="3">2126.10624</td><td rowspan="3">4.97E+06</td><td rowspan="3">1045.02603</td><td rowspan="3">0.007621</td></tr>
<tr><td rowspan="2">[M+K]+</td><td rowspan="2">1083.5126</td><td rowspan="2">1083.51324</td><td rowspan="2">0.00063</td><td rowspan="2">7.09E+05</td></tr>
<tr></tr>

<tr><td rowspan="6">16</td><td rowspan="3">9+</td><td rowspan="3">2246.15981</td><td rowspan="3">1.54E+09</td><td rowspan="3">2366.04801</td><td rowspan="3">1.11E+07</td><td rowspan="3">1078.99381</td><td rowspan="3">0.007179</td><td rowspan="6">0.8</td><td rowspan="2">[M+H]+</td><td rowspan="2">1080.60275</td><td rowspan="2">1080.60264</td><td rowspan="2">-0.00011</td><td rowspan="2">3.48E+05</td><td rowspan="6">1079.27</td></tr>
<tr></tr>
<tr><td rowspan="2">[M+Na]+</td><td rowspan="2">1102.58469</td><td rowspan="2">1102.58469</td><td rowspan="2">0</td><td rowspan="2">1.71E+07</td></tr>
<tr><td rowspan="3">10+</td><td rowspan="3">2021.60364</td><td rowspan="3">6.32E+08</td><td rowspan="3">2129.57833</td><td rowspan="3">6.35E+06</td><td rowspan="3">1079.74684</td><td rowspan="3">0.010056</td></tr>
<tr><td rowspan="2">[M+K]+</td><td rowspan="2">1118.55863</td><td rowspan="2">1118.55785</td><td rowspan="2">-0.00078</td><td rowspan="2">1.42E+06</td></tr>
<tr></tr>

<tr><td rowspan="6">2</td><td rowspan="3">9+</td><td rowspan="3">2246.17168</td><td rowspan="3">1.07E+09</td><td rowspan="3">2380.31615</td><td rowspan="3">4.50E+06</td><td rowspan="3">1207.3003</td><td rowspan="3">0.00422</td><td rowspan="6">0.59</td><td rowspan="2">[M+H]+</td><td rowspan="2">1208.61277</td><td rowspan="2">1208.61291</td><td rowspan="2">0.00014</td><td rowspan="2">3.35E+05</td><td rowspan="6">1207.6</td></tr>
<tr></tr>
<tr><td rowspan="2">[M+Na]+</td><td rowspan="2">1230.59471</td><td rowspan="2">1230.59471</td><td rowspan="2">0</td><td rowspan="2">1.26E+07</td></tr>
<tr><td rowspan="3">10+</td><td rowspan="3">2021.60364</td><td rowspan="3">6.52E+08</td><td rowspan="3">2142.3817</td><td rowspan="3">5.70E+06</td><td rowspan="3">1207.78061</td><td rowspan="3">0.008736</td></tr>
<tr><td rowspan="2">[M+K]+</td><td rowspan="2">1246.56865</td><td rowspan="2">1246.56867</td><td rowspan="2">2.01E-05</td><td rowspan="2">1.17E+06</td></tr>
<tr></tr>

<tr><td rowspan="6">11</td><td rowspan="3">9+</td><td rowspan="3">2246.21915</td><td rowspan="3">3.97E+08</td><td rowspan="3">2317.19517</td><td rowspan="3">2.39E+06</td><td rowspan="3">638.78422</td><td rowspan="3">0.00603</td><td rowspan="6">0.57</td><td rowspan="2">[M+H]+</td><td rowspan="2">635.30676</td><td rowspan="2">-</td><td rowspan="2">-</td><td rowspan="2">-</td><td rowspan="6">638.59</td></tr>
<tr></tr>
<tr><td rowspan="2">[M+Na]+</td><td rowspan="2">657.2887</td><td rowspan="2">657.2887</td><td rowspan="2">0</td><td rowspan="2">5.13E+06</td></tr>
<tr><td rowspan="3">10+</td><td rowspan="3">2021.59403</td><td rowspan="3">1.80E+08</td><td rowspan="3">2085.44042</td><td rowspan="3">9.28E+05</td><td rowspan="3">638.46389</td><td rowspan="3">0.005155</td></tr>
<tr><td rowspan="2">[M+K]+</td><td rowspan="2">673.26264</td><td rowspan="2">673.23264</td><td rowspan="2">-0.03</td><td rowspan="2">4.59E+05</td></tr>
<tr></tr>

<tr><td rowspan="6">15</td><td rowspan="3">9+</td><td rowspan="3">2246.25476</td><td rowspan="3">9.94E+08</td><td rowspan="3">2327.13995</td><td rowspan="3">4.96E+06</td><td rowspan="3">727.96671</td><td rowspan="3">0.004991</td><td rowspan="6">0.48</td><td rowspan="2">[M+H]+</td><td rowspan="2">729.22253</td><td rowspan="2">-</td><td rowspan="2">-</td><td rowspan="2">-</td><td rowspan="6">728.7</td></tr>
<tr></tr>
<tr><td rowspan="2">[M+Na]+</td><td rowspan="2">751.20447</td><td rowspan="2">751.20447</td><td rowspan="2">0</td><td rowspan="2">8.15E+06</td></tr>
<tr><td rowspan="3">10+</td><td rowspan="3">2021.61325</td><td rowspan="3">6.75E+08</td><td rowspan="3">2094.54405</td><td rowspan="3">3.02E+06</td><td rowspan="3">729.30793</td><td rowspan="3">0.004473</td></tr>
<tr><td rowspan="2">[M+K]+</td><td rowspan="2">767.17841</td><td rowspan="2">767.17837</td><td rowspan="2">-4.08E-05</td><td rowspan="2">2.48E+06</td></tr>
<tr></tr>

<tr><td rowspan="6">15</td><td rowspan="3">9+</td><td rowspan="3">2246.25476</td><td rowspan="3">9.94E+08</td><td rowspan="3">2335.21901</td><td rowspan="3">4.33E+06</td><td rowspan="3">800.67825</td><td rowspan="3">0.004359</td><td rowspan="6">0.45</td><td rowspan="2">[M+H]+</td><td rowspan="2">802.50162</td><td rowspan="2">802.50122</td><td rowspan="2">-0.0004</td><td rowspan="2">4.43E+05</td><td rowspan="6">801.01</td></tr>
<tr></tr>
<tr><td rowspan="2">[M+Na]+</td><td rowspan="2">824.48356</td><td rowspan="2">824.48356</td><td rowspan="2">0</td><td rowspan="2">9.55E+06</td></tr>
<tr><td rowspan="3">10+</td><td rowspan="3">2021.61325</td><td rowspan="3">6.75E+08</td><td rowspan="3">2101.77215</td><td rowspan="3">3.25E+06</td><td rowspan="3">801.58893</td><td rowspan="3">0.00482</td></tr>
<tr><td rowspan="2">[M+K]+</td><td rowspan="2">840.4575</td><td rowspan="2">840.45719</td><td rowspan="2">-0.00031</td><td rowspan="2">1.17E+06</td></tr>
<tr></tr>

<tr><td rowspan="6">1</td><td rowspan="3">9+</td><td rowspan="3">2246.15981</td><td rowspan="3">2.19E+09</td><td rowspan="3">2366.07435</td><td rowspan="3">6.83E+06</td><td rowspan="3">1079.23084</td><td rowspan="3">0.003127</td><td rowspan="6">0.4</td><td rowspan="2">[M+H]+</td><td rowspan="2">1080.60267</td><td rowspan="2">-</td><td rowspan="2">-</td><td rowspan="2">-</td><td rowspan="6">1079.27</td></tr>
<tr></tr>
<tr><td rowspan="2">[M+Na]+</td><td rowspan="2">1102.58461</td><td rowspan="2">1102.58461</td><td rowspan="2">0</td><td rowspan="2">8.77E+05</td></tr>
<tr><td rowspan="3">10+</td><td rowspan="3">2021.69017</td><td rowspan="3">6.31E+08</td><td rowspan="3">2129.68501</td><td rowspan="3">4.46E+06</td><td rowspan="3">1079.94843</td><td rowspan="3">0.007075</td></tr>
<tr><td rowspan="2">[M+K]+</td><td rowspan="2">1118.55855</td><td rowspan="2">-</td><td rowspan="2">-</td><td rowspan="2">-</td></tr>
<tr></tr>

<tr><td rowspan="6">5</td><td rowspan="3">9+</td><td rowspan="3">2246.06487</td><td rowspan="3">5.70E+08</td><td rowspan="3">2317.85214</td><td rowspan="3">2.35E+06</td><td rowspan="3">646.08548</td><td rowspan="3">0.004125</td><td rowspan="6">0.39</td><td rowspan="2">[M+H]+</td><td rowspan="2">651.81708</td><td rowspan="2">-</td><td rowspan="2">-</td><td rowspan="2">-</td><td rowspan="6">645.67</td></tr>
<tr></tr>
<tr><td rowspan="2">[M+Na]+</td><td rowspan="2">673.79902</td><td rowspan="2">673.79902</td><td rowspan="2">0</td><td rowspan="2">4.10E+06</td></tr>
<tr><td rowspan="3">10+</td><td rowspan="3">2021.55557</td><td rowspan="3">5.91E+08</td><td rowspan="3">2086.17726</td><td rowspan="3">2.17E+06</td><td rowspan="3">646.21685</td><td rowspan="3">0.003671</td></tr>
<tr><td rowspan="2">[M+K]+</td><td rowspan="2">689.77296</td><td rowspan="2">-</td><td rowspan="2">-</td><td rowspan="2">-</td></tr>
<tr></tr>

<tr><td rowspan="6">16</td><td rowspan="3">9+</td><td rowspan="3">2246.15981</td><td rowspan="3">1.54E+09</td><td rowspan="3">2426.30282</td><td rowspan="3">4.16E+06</td><td rowspan="3">1621.28713</td><td rowspan="3">0.002703</td><td rowspan="6">0.33</td><td rowspan="2">[M+H]+</td><td rowspan="2">1622.72335</td><td rowspan="2">1622.72335</td><td rowspan="2">0</td><td rowspan="2">3.58E+07</td><td rowspan="6">1620.67</td></tr>
<tr></tr>
<tr><td rowspan="2">[M+Na]+</td><td rowspan="2">1644.70529</td><td rowspan="2">1644.70479</td><td rowspan="2">-0.00051</td><td rowspan="2">3.80E+06</td></tr>
<tr><td rowspan="3">10+</td><td rowspan="3">2021.60364</td><td rowspan="3">6.32E+08</td><td rowspan="3">2183.76103</td><td rowspan="3">3.04E+06</td><td rowspan="3">1621.5739</td><td rowspan="3">0.004806</td></tr>
<tr><td rowspan="2">[M+K]+</td><td rowspan="2">1660.67923</td><td rowspan="2">1660.6709</td><td rowspan="2">-0.00834</td><td rowspan="2">4.11E+06</td></tr>
<tr></tr>
</table>

**Code S1. Python script for automated analysis of integrated dual-mode native MS datasets.**

```
# Code S1. Native MS binding ratio and CIAS MS adduct confirmation workflow
# Local file paths and export commands are omitted for clarity.

import re
import numpy as np
import pandas as pd

PROTON = 1.007276466812
ADDUCTS = {
    "H": PROTON,
    "Na": 22.989218,
    "K": 38.963158,
}

# Native MS parameters used for the reproduced binding ratio.
IP_INCLUDE_ACETATE = False       # Ip uses I0 protein peak only
TOP_N_CHARGES = 2                # binding ratio uses top 2 protein charge states
MATCH_TOL_PROTEIN = 1.0          # Da, protein peak matching
MATCH_TOL_COMPLEX = 0.1          # Da, Native MS complex peak assignment

# CIAS MS parameters used for the previous matching logic.
CIAS_SEARCH_TOL_DA = 15.0        # broad Native-derived adduct window
CIAS_CONFIRM_TOL_DA = 0.2        # final H/Na/K confirmation around inferred neutral mass
TOP_N_GLOBAL = 3                 # top CIAS peaks retained across H/Na/K windows


def binding_ratio(total_complex_intensity, protein_intensity_sum):
    """Native MS ligand binding ratio used in Protein-binding ligands.xlsx."""
    denom = protein_intensity_sum + total_complex_intensity
    return total_complex_intensity / denom if denom > 0 else np.nan


def per_charge_ligand_mass(protein_mz, complex_mz, charge):
    """Observed ligand mass from one Native MS charge state."""
    return (complex_mz - protein_mz) * charge


def native_delta_mass_from_top2(row):
    """
    Intensity-weighted observed Native MS delta mass from the top two charge states.
    This value is used as the Native-derived neutral mass for CIAS MS searching.
    """
    masses = []
    weights = []
    for suffix in ["z1", "z2"]:
        z = row.get(suffix)
        protein_mz = row.get(f"protein_mz_found_{suffix}")
        complex_mz = row.get(f"complex_found_mz_{suffix}")
        complex_intensity = row.get(f"Ic_{suffix}", 1.0)
        if pd.notna(z) and pd.notna(protein_mz) and pd.notna(complex_mz):
            masses.append(per_charge_ligand_mass(protein_mz, complex_mz, z))
            weights.append(complex_intensity if pd.notna(complex_intensity) and complex_intensity > 0 else 
1.0)
    return np.average(masses, weights=weights) if masses else np.nan


def select_visible_filtered_rows(native_results, filtered_reference_rows):
    """
    Use the analyst-curated Excel filtered rows as the final candidate set.
    Rows are matched by Native MS file, ligand name, and ligand mass.
    """
    def key(df):
        return (
            df["file"].astype(str) + "|" +
            df["ligand_name"].astype(str) + "|" +
            pd.to_numeric(df["ligand_mass"], errors="coerce").round(6).astype(str)
        )

    native_results = native_results.copy()
    filtered_reference_rows = filtered_reference_rows.copy()
    native_results["row_key"] = key(native_results)
    filtered_reference_rows["row_key"] = key(filtered_reference_rows)
    filtered_reference_rows["filtered_order"] = range(len(filtered_reference_rows))
```

```python
    selected = native_results.merge(
        filtered_reference_rows[["row_key", "filtered_order"]],
        on="row_key",
        how="inner",
    )
    selected = selected.sort_values("filtered_order").drop(columns=["row_key", "filtered_order"])
    return selected.reset_index(drop=True)


def peaks_in_window(spectrum, target_mz, tolerance_da):
    """Return all CIAS MS peaks within target_mz +/- tolerance_da."""
    mz = spectrum["mz"]
    intensity = spectrum["intensity"]
    keep = np.abs(mz - target_mz) <= tolerance_da
    return pd.DataFrame({"mz": mz[keep], "intensity": intensity[keep]}).sort_values(
        ["intensity", "mz"], ascending=[False, True]
    )


def choose_cias_peak_by_previous_logic(spectrum, native_delta_mass):
    """
    Previous CIAS MS matching logic:
    1. Build H, Na, and K target m/z values from the Native MS observed delta mass.
    2. Collect all CIAS peaks within +/-15 Da of those three target windows.
    3. Keep the global top 3 peaks by intensity.
    4. Select the top-3 peak with the smallest absolute m/z error to any H/Na/K target.
    """
    all_peaks = []
    targets = {adduct: native_delta_mass + shift for adduct, shift in ADDUCTS.items()}

    for adduct, target_mz in targets.items():
        peaks = peaks_in_window(spectrum, target_mz, CIAS_SEARCH_TOL_DA)
        if not peaks.empty:
            peaks = peaks.copy()
            peaks["window_adduct"] = adduct
            peaks["target_mz"] = target_mz
            peaks["dmz_to_window_target"] = peaks["mz"] - target_mz
            all_peaks.append(peaks)

    if not all_peaks:
        return None

    top_peaks = pd.concat(all_peaks, ignore_index=True).sort_values(
        ["intensity", "mz"], ascending=[False, True]
    ).head(TOP_N_GLOBAL)

    evaluated = []
    for _, peak in top_peaks.iterrows():
        dmz_by_adduct = {adduct: peak["mz"] - target_mz for adduct, target_mz in targets.items()}
        best_adduct = min(dmz_by_adduct, key=lambda adduct: abs(dmz_by_adduct[adduct]))
        evaluated.append({
            "chosen_peak_mz": peak["mz"],
            "chosen_peak_intensity": peak["intensity"],
            "chosen_peak_best_target": best_adduct,
            "chosen_peak_dmz_to_best_target": dmz_by_adduct[best_adduct],
        })

    return min(evaluated, key=lambda x: abs(x["chosen_peak_dmz_to_best_target"]))


def reinterpret_chosen_cias_peak(chosen_peak_mz, same_well_cias_ligands):
    """
    Reinterpret the same chosen CIAS peak as H, Na, or K.
    The retained interpretation is the one whose inferred neutral mass is nearest
    to a same-well CIAS ligand database entry.
    """
    candidates = []
    for adduct, shift in ADDUCTS.items():
        neutral_mass = chosen_peak_mz - shift
        mass_errors = same_well_cias_ligands["mass"] - neutral_mass
        best_idx = mass_errors.abs().idxmin()
        candidates.append({
            "best_reinterpreted_adduct": adduct,
            "cias_inferred_neutral_mass": neutral_mass,
            "cias_nearest_ligand": same_well_cias_ligands.loc[best_idx, "ligand_name"],
            "cias_nearest_ligand_mass": same_well_cias_ligands.loc[best_idx, "mass"],
            "cias_nearest_mass_error_Da": mass_errors.loc[best_idx],
        })
    return min(candidates, key=lambda x: abs(x["cias_nearest_mass_error_Da"]))
```

```python
def confirm_h_na_k_adducts(spectrum, inferred_neutral_mass):
    """
    After the CIAS neutral mass is inferred, report the H, Na, and K adduct evidence.
    The reported peak is the highest CIAS peak within +/-0.2 Da of inferred_mass + adduct.
    """
    confirmations = {}
    for adduct, shift in ADDUCTS.items():
        target_mz = inferred_neutral_mass + shift
        peaks = peaks_in_window(spectrum, target_mz, CIAS_CONFIRM_TOL_DA)
        if peaks.empty:
            confirmations[adduct] = {
                "theoretical_mz": target_mz,
                "observed_mz": np.nan,
                "mz_error_Da": np.nan,
                "intensity": np.nan,
                "detected": False,
            }
        else:
            best = peaks.iloc[0]
            confirmations[adduct] = {
                "theoretical_mz": target_mz,
                "observed_mz": best["mz"],
                "mz_error_Da": best["mz"] - target_mz,
                "intensity": best["intensity"],
                "detected": True,
            }
    return confirmations


def keep_native_cias_consistent_matches(results):
    """
    Final table rule for the supporting information:
    keep only records where the Native MS ligand ID agrees with the nearest CIAS ligand ID.
    """
    return results[results["ligand_name"].astype(str) == results["cias_nearest_ligand"].astype(str)].copy()


def analysis_workflow(native_results, filtered_reference_rows, cias_spectra_by_well, cias_ligands_by_well):
    """High-level workflow used to produce the final supporting-information table."""
    selected = select_visible_filtered_rows(native_results, filtered_reference_rows)
    output_rows = []

    for _, row in selected.iterrows():
        well = row["well"]
        spectrum = cias_spectra_by_well[well]
        same_well_ligands = cias_ligands_by_well[well]

        native_delta_mass = native_delta_mass_from_top2(row)
        chosen = choose_cias_peak_by_previous_logic(spectrum, native_delta_mass)
        if chosen is None:
            continue

        reinterpretation = reinterpret_chosen_cias_peak(chosen["chosen_peak_mz"], same_well_ligands)
        adduct_confirmations = confirm_h_na_k_adducts(
            spectrum,
            reinterpretation["cias_inferred_neutral_mass"],
        )

        output_rows.append({
            **row.to_dict(),
            "native_delta_mass_for_cias": native_delta_mass,
            **chosen,
            **reinterpretation,
            "H_adduct": adduct_confirmations["H"],
            "Na_adduct": adduct_confirmations["Na"],
            "K_adduct": adduct_confirmations["K"],
        })

    return keep_native_cias_consistent_matches(pd.DataFrame(output_rows))
```
